# Illinois State Medical Society; Annual Meeting

**Published:** 1887-06

**Authors:** 


### SOCIETY REPORTS.

### ILLINOIS STATE MEDICAL SOCIETY.

[*Reported for The Chicago Medical Journal and Examiner by Dr. J. L. Gray.*]

The Thirty-seventh annual meeting of the Illinois State Medical Society was called to order at 10:30 A. M., May 17th, Vice-President Dr. Elias Wenger in the chair.

Dr. Wenger, in opening the session, spoke of the death of the President, Dr. W. T. Kirk, of Atlanta.

Dr. E. Ingals, Chairman of the Committee of Arrangements, reported that, in consequence of the unsettled state of railroad matters, the committee had been unable to get any reduction in fare for those attending this meeting of the Society. The committee recommended that the hours for meeting be at 9 A. M. and 2 P. M., and for adjournment at 12 M. and 6 P. M., also that the order of business shall be such as is printed on the programme.

Dr. J. L. White, of Bloomington, said: Mr. President, I crave your indulgence, at this time, for a few minutes.

This is the first time, so far as I know, in the history of this Society that it has been called upon to mourn the death of its President, and it seems to me eminently proper that we pause at this early stage in our proceedings to pay a slight tribute to his memory.

Dr. William Thomas Kirk was born Oct. 27, 1833. At the age of eighteen he commenced the study of medicine and graduated in 1854 from the Louisville University. After graduation he came to Illinois, locating at Farmer City, remaining one year, thence removing to Atlanta, where his death took place March 25th. Dr. Kirk was the victim of fatty degeneration of the heart.

In closing, Dr. White suggested that the society send resolutions of condolence to the family of the deceased President, and as a mark of respect that during the sessions of the society the President's chair be appropriately draped. The suggestion was put by Dr. White in the form of a motion, which was seconded by Dr. W. T. Montgomery, and unanimously adopted by the society.

On motion of Dr. E. Ingals the report of Dr. White was referred to the Committee on Publication to appear in the Transactions, under the Necrological Report.

Dr. S. C. Plummer, of Rock Island, moved and Dr. R. Tilley, of Chicago, seconded the motion that the report of the Committee of Arrangements be adopted. Carried unanimously.

Dr. A. Reeves Jackson delivered the address of welcome on behalf of the profession of Chicago, expressing great pleasure at once again being able to bid the representatives of the medical profession of Illinois a welcome to the city.

On behalf of the Society, Dr. E. P. Cook, of Mendota responded. He said: When I was informed that it would be expected of me to talk back to the *Doctor* of "*Innocents Abroad*," it occurred to me that, Mark-Twain-like, I had better make my arrangements to speak extemporaneously. The doctor then proceeded to read from manuscript, aptly drawing comparisons between the city and country doctor. He was proud of the fact that Chicago has become a medical educational center second to none in our country. "We of the suburban fraternity, compared to Chicago, can think of some things the doing of which would be mutually beneficial, and some we hope you won't do. Don't permit any more medical college enterprises to spring up. Don't make any further subdivisions of the field of medicine. Don't accept material out of which to make doctors if it is found defective, either as to capacity or preliminary education.

Some queries we would like to propound are: In what proportion of cases of carcinoma of the stomach should we resort to resection?

What surgical interference is justifiable in valvular disease of the heart?

How to locate abscesses of the brain?

How can we destroy bacteria without destroying the unfortunate one infested with them?"

The Secretary read letters of regret from Drs. D. S. Booth, of Sparta, and C. Goodbrake, of Clinton.

Dr. J. H. Hollister moved that the call of the committees be gone through and that the report of the Committee on the Practice of Medicine be received at 2 o'clock, which was lost.

Dr. Tilley moved that the printed order be followed out. Carried.

In the case of committees, all except those on Drugs and Medicines, Diseases of Children and Hydrophobia were announced as being ready to report.

On motion of Dr. E. Ingals, Dr. L. S. Tesson, Surgeon U. S. Army, and Dr. James G. Kiernan, of Chicago, were made members by invitation.

### REPORT OF THE COMMITTEE ON PRACTICE OF MEDICINE.

The first scientific paper was that of Dr. D. R. Brower, Chairman of the Committee on the Practice of Medicine. He said since the last meeting of the State Medical Society there has been throughout the whole domain of medicine remarkable activity. Many new and important additions have been made to the treatment of disease, to a few of which he proposed to call attention.

The treatment of phthisis by rectal injection of sulphurous acid gas, communicated to the Academy of Sciences, at Paris, by M. Bergeon, is one of the most important of these discoveries. This measure is based upon the physiological fact established by Claude Bernard that the introduction of toxic substances by the rectum does not offer so much danger as when introduced by the mouth, so long as pulmonary elimination is not interfered with.

The results of applying this method in phthisis are:

First: After a few days, diminution of cough. Second: A profound modification of the quantity and quality of expectoration. Third: The suppression of sweating and gradual disappearance of moist *râles*, and improvement of the general condition.

The next subject to which he called attention is contained in a paper by Dr. John D. Skeer, of Chicago, entitled "A Pathognomonic Symptom in Diagnosis of Tubercular Meningitis," the symptom being a small circle which forms in the iris near to and completely surrounding the pupillary margin. When it begins to appear it is very distinct, and resembles a wreath of thin white clouds, the edge of which extends at first to the free border of the iris; in from 12 to 36 hours the whole margin of the iris will be involved, having become of a whitish or yellowish brown color, and appearing irregular, thickened, and somewhat granular. These changes are commenced in both eyes simultaneously. This cloud-like appearance is in some cases very evanescent; hence the necessity of examining the iris at every visit. These rings are permanent, and grow narrower and larger with the progressive dilatation of the pupils as the disease advances.

Much has been written on the subject of antipyretics, the latest discovery in this direction being antifibrin. In general terms it may be stated that it is about four times the strength of antipyrin. The effect of the drug upon the temperature is noticed in about an hour.

Many valuable contributions will be found in current literature on the relief of insomnia by the new hypnotics, especially urethan and paraldehyde. Experience teaches that urethan should be given in large doses.

On motion of Dr. E. Ingals, the Society adjourned to meet at 2 P.M.

---

### TUESDAY AFTERNOON SESSION.

The Society was called to order at 2.20 P.M., Vice-President Wenger in the chair.

Dr. D. R. Brower gave a short resume of the balance of the report on the Practice of Medicine, after which Dr. P. H. Oyler of Mt. Pulaski, of the same committee, read a paper on "Diphtheria," in which he claimed that true diphtheria is a zymotic disease, which he believes to be undeniable, and that every theory to the contrary must fall to the earth. That it attacks only unhealthy mucous membranes, and through them finds entrance into the circulation, as claimed by some, he believes to be groundless. He said he had not investigated chemically the character of the virus, but from observation and his convictions, he based his treatment upon somewhat different reasoning than that almost universally accepted. No one will deny that cholera is a zymotic disease. He had been taught that in cholera there existed as uper-acidity of the sanguineous fluids, and he believes that in *all* zymotic diseases the same condition exists to a greater or less degree. Taking the view that there is an acid condition in the development of the disease, the remedial agent sought for should be the one that would meet the greatest number of indications. The treatment he had been using since 1880 is carbonate of ammonia, jaborandi, benzoate of soda and quinia sulphate, steam and poultices around the throat. In his experience this treatment had been much more successful than Tr. Ferri. Chlorid. Carbonate of ammonia had been given in large doses, 30 to 60 grains in solution.

Dr. E. F. Ingals, in opening the discussion upon the gaseous treatment of tuberculosis, said he could not look upon that method of treatment as a "cure all," by any means. In all the cases in which he had used that treatment he had continued the other treatment the same as if the new method had not been used. He had seen the temperature go down within four or five days, the expectoration gradually diminish, and the night-sweats cease. However, in one case the temperature had gone up again, and things looked bad. In one case the laryngeal symptoms were made worse. It is to be remembered that there are dangers to be guarded against in administering the gas. As a rule, for a time at least, the injections should be given by the physician.

He had been much interested in the paper on diphtheria. The treatment suggested by Dr. Oyler seemed to him novel at least. He had found, however, that every one has his peculiar treatment, which is peculiarly successful. He had no doubt that some of those present had treated hundreds of cases of diphtheria, with few deaths. But all know that severe cases of diphtheria are very likely to die. The old-fashioned treatment has seemed to him to be more useful. Certainly nothing should be done to increase the struggling of the patient.

Dr. J. A. Robison said that his experience in making the sulphurous waters had been such that he gives preference to the natural mineral waters. He had been unable to get the chemical reactions with the artificial waters; but he did not mention the mineral water which he generally uses because he did not wish to seem to be advertising it.

Dr. N. S. Davis said he had very few words to say in reference to this method of treatment of consumption, for he had no personal experience with it, but he occasionally gets a little history of some of the cases thus treated. A young man in the suppurative stage of consumption came to his office. He had been using the treatment ten days, and, according to his report, instead of diminishing the suppuration, he insisted that his expectoration had increased, and that he had received no benefit whatever. He had only used it ten days, and Dr. Davis encouraged him to continue its use. He mentioned this simply to show that in diminishing the night-sweats, expectoration, etc., that case had not been in accordance with the usual results.

He said he rose more especially to ask whether, in devising treatment of diphtheria or any other acute febrile disease, we are not too apt to confine our efforts to the finding of some remedy or some combination of remedies that are to be commenced and continued through to the end. One man advocates the tincture of iodine as the best remedy; another carbonate of ammonia in big or little doses, whilst another administers calomel from the beginning to the end, and all for the same purpose. Each one has some leading remedy, and something to prop it up with, as he goes along. It had seemed to him, in his experience in this disease, that there is a definite process, general conditions, conditions of secretion, conditions of exudation—products essentially different as they progress. He had attended many cases of diphtheria in former times, and had gone through many epidemics. Epidemics differ in their severity, in their fatality, their amenability to treatment, as clearly as the differences in temperature.

When he came to a case of diphtheria at its beginning, he found, most frequently, a dry skin and a tendency to the checking of secretions generally. After the lapse of four or five days the exudation, not only on the membranes, but the glands of the neck become tumefied and reach their culminating point. In the course of twenty-four hours, if the patient lives, one can distinguish signs of disintegration, gradual softening and breaking down of the tissues, the pulse more rapid, easily compressible, and small ulcers appear in the membrane. In the first stage some remedies may be used to promote needful secretion and hold in check the excessive temperature. If there is any stage at which calomel is wanted it is at that time. But if iron and carbonate of ammonia are commenced at that stage, usually no good results follow.

But just as soon as the culminating stage is reached and the pulse begins to be softer and quicker,—when the countenance is a little less florid—then begins the stage of depression, and then is the time for the use of carbonate of ammonia, iron, or anything that will act in the same direction. The point that he wished to make is, that we must adjust our remedies to the stage of the disease, and not begin with one remedy and carry it through the successive stages with little or no heed as to the indications.

Dr. D. A. K. Steele said in conclusion, that just at present Bergeon's method is the live subject of the day, and, while it is not applicable to the cure of consumption, and does not destroy the bacilli, yet it is of benefit when it comes in contact with any suppurating surface. After the symptoms of sepsis or blood-poisoning have disappeared, to a certain extent it is of advantage as a germicide, that is, in lessening the quantity of the sputum and its offensiveness. But it does not cure the disease. It is the fashion at present, and we have to use it. The apparatuses that are on sale are very frail and quite expensive. One can be made very easily for four or five dollars. In regard to the usefulness of this method of treatment he would say that it is simply on trial and that he is not an enthusiast in its advocacy, and would venture the prediction that it will prove quite evanescent.

Dr. E. Ingals said he was reminded by the discussion of these subjects of the old proverb, "A new broom sweeps clean," to which Dr. N. S Davis replied, that it may soon get worn and soiled.

Dr. G. L. Corcoran said he had heard a great deal said in regard to this new treatment, the injecting of gases in pulmonary phthisis. In all probability there may be something in it. There must have been something to start the matter, and the probability is that something may be evolved from it. It might seem improbable to those who had long been engaged in the practice of medicine that the injection of gases would benefit tubercle in the lung. But nevertheless, stranger things have come to pass, and it may be that by prosecuting this matter and using different gases, some thing of importance may be arrived at. In the treatment of phthisis pulmonalis he thought that he had found nothing so beneficial as the use of iron and arsenic. There being no further discussion, the papers were referred to the Committee on Publication.

### REPORT OF THE COMMITTEE ON SURGERY.

Dr. D. A. K. Steele, of Chicago, in making the report of the Committee on Surgery, said he would give a resumé of the principal work done during the year by those who may be regarded as leaders in the science and art of surgery. The elaborate memoir of Dr. N. Senn, of Milwaukee, on "The Surgery of the Pancreas," represents the greatest amount of original research during the year in this country, and supplies the data upon which to base rational methods of treatment for such diseases or injuries of the pancreas as may be amenable to surgical interference.

OSTEO-MYELITIS.—The micro-organisms of acute infecting osteomyelitis have been identified, according to Rosenbech, Becker and Garre, as Staphylococcus Pyogenus Aureus, and Staphylococcus Pyogenus Albus, and Streplococcus Pyogeus. Ooccasionally the same micro-organisms are found in pyæmic actinomycosis.

The parasite of this disease was first described by Lebert in 1857, and Dr. W. T. Belfield, of Chicago, was the first to identify the disease in cattle known as lumpy-jaw as actinomycosis, from the ever-present millet-seed bodies which, under the microscope, are shown to be fungus mycelia of the first class. The only efficient treatment is thorough opening and scraping of the abscess-cavity, with destruction of the infected tissues.

Malignant pustule retains its interest, as it was the first mycotic disease discovered in man.

TUBERCULOSIS.—During the past few years scrofulous, strumous, malignant diseases, white-swelling and lupus have been definitely classified as tuberculosis of bones, joints or synovial surfaces, and rendered curable by the recognition of their pathology.

WHITE THROMBUS.—It has been shown that foreign substances in the blood-currents are soon covered with a blood-plaque. When stasis from any cause occurs, the third corpuscle or blood-plaque undergoes a vicious transformation, adhering to the white corpuscle and the vessel-wall, causing a white thrombus.

ANTISEPTICS IN SURGERY.—Dr. Knapp, of New York, after a careful series of experiments, believes that suppuration is always caused by bacteria, and has proven that sterilized solutions of chemical irritants introduced into the blood are not followed by suppuration. He concludes that fermentation is the decomposition of carbo-hydrates into simpler compounds, by the agency of living microbes.

THORACO-PLASTY.—Has been performed, with the result of obliterating old pleural suppurating cavities.

SURGERY OF THE KIDNEYS.—Surgical operations on the kidneys are becoming so frequent as to require no special notice. The operations usually done are:

Nephrorrhaphy or suturing a floating kidney.
2. Nephrotomy or exploratory incision.
3. Nephrectomy or excision of a diseased kidney.

SUPRA-PUBIC CYSTOTOMY.—Has been gaining favor again.

WOUND-HEALING.—In the reporter's private practice, the matter of antiseptics is thoroughly looked after and solutions of bichloride, 1 to 500 freely used in washing the field of operation. The spray has been entirely abandoned by him during the last three years.

Dr. Steele reports several cases operated on by himself, three of shoulder, one of thigh, two of leg amputations and one of excision of the hip. In operations on bone, he follows Schedo's method. In simple fractures, he uses a primary plaster-cast, while in delayed union or ununited fractures he drills the unossified band of callus between the fragments in various directions. If this fails, he cuts down upon the ends and fastens them firmly with bone-pegs.

Dr. B. F. Crummer, of Warren, reported a case of unique wound of the chest, in which the lung was extensively lacerated, and a large hemorrhage occurred into the right thorax. No air could gain access through the wound and no foreign body remained in the chest. He also reported several cases of pleuritic effusion and one of spontaneous recovery from empyema.

By vote of the society, discussion of the report on surgery was made the special order for Wednesday at 2 o'clock, the discussion not to exceed one hour.

REPORT OF THE COMMITTEE ON OBSTETRICS.

Dr. Ellen A. Ingersoll, of Canton, Chairman of the Committee on Obstetrics, gave a general resumé of the literature on the subject, and considered especially

THE SIGNS OF PREGNANCY.—During the first eight weeks of pregancy the changes in the uterus are practically limited to the body of the organ. As a result of these changes the uterine body loses its pear shape. These changes constitute Hegar's sign, viz.: "The increase in the anterior curvature of the uterus, with increased elasticity of the uterine walls."

MANAGEMENT OF THE SECUNDINES.—The management of abortions, attended with retained secundines, has been much and ably discussed, the preponderance of expression being to favor the immediate removal of the uterine contents when the ovum had been expelled, and the placenta and membranes remained. In the latter months of pregnancy, the speaker knew of no facts which justify the expectant plan in the management of the third stage of labor.

ANTISEPTICS.—While there are none who do not believe in aseptic principles there are many who do not seem to realize that absolute cleanliness is the great antiseptic precaution. The literature during the year, pro and con, was elaborately discussed.

FORCEPS.—In the review of the literature, mention was made of Breus' and Felsenreich's modification of Tarnier's and Dr. Brooks F. Wells' device for converting an ordinary obstetrical forceps into an axis-traction forceps, and the pelvic-outlet forceps of Dr. A. J. Stone.

PUERPERAL ECLAMPSIA.—The three most important principles to be carried out in the treatment of puerperal eclampsia are sedation or suspension of the reflex functions of the spinal cord; prompt reduction of arterial pressure, and the elimination of effete matter from the system. Veratrum viridi probably stands first as a sedative.

Dr. T. G. Thomas, in the strongest manner, advises the induction of premature labor in eclampsia occurring at the sixth, seventh and eighth months of pregnancy.

EXRA-UTERINE PREGNANCY.—As compared with other abnormalities of pregnancy, abdominal gestation is rare. Ectopic gestation falls naturally into three groups: First, cases seen in the early months; here the placental souffle can be heard with the vaginal stethescope at the end of the sixth week. Second, cases at mid-term; here if rupture occurs death is almost certain, therefore operation should always be undertaken. Third, cases after the seventh month where the child is living; in these cases cæsarian section is indicated, and operation is a duty. The use of electricity in this disease is at least receiving the attention it merits.

DYSTOCIA AND PREMATURE LABOR.—The past few years have been especially fruitful in measures for preventing and overcoming severe dystocia, one of the causes of which is lateroversion at the beginning of labor.

In conclusion, Dr. Ingersoll gave a resumè of the latest information on cæsarian section, giving in brief the modification of Poro's operation devised by Dr. John Bartlett, of Chicago.

"Does a Retention of the Secundines Necessitate Septic Poisoning?" is the title of the

SUPPLEMENTARY REPORT

of Dr. W. H. Conibear, one of the Committee on Obstetrics. The paper was read by Dr. Ellen A. Ingersoll, in the absence of the author. The writer of the paper is of the opinion that retention of the secundines is not a frequent cause of septic poisoning, and cites several cases from his personal experience in support of his doctrine.

Dr. C. W. Earle, in opening the discussion of the preceding papers, called attention to the fact that Hegar's sign was long since recognized, but no one until Hegar had considered it with sufficient definiteness to describe it. Another symptom or condition of importance in the diagnosis of pregnancy is the discoloration of the mucous membrane of the vagina. This has been brought forward as a new symptom within the past few months, but it is nevertheless old. Doctor Earle said that the facts noted in the report of the long retention of secundines simply demonstrated that so long as no septic matter came to the woman, these foreign bodies were practically inert. In Italy, or in any of the European countries, if an operator is asked, after one of the severer obstetric operations, "Will that woman recover?" he will answer "Yes, she will recover if I have not infected her." Are we, as American physicians, ready to take the responsibility of thus negligently infecting puerperal women? He said that this was a question which needed the most careful discussion.

Dr. O. B. Will, of Peoria, said he would like to enter his most emphatic protest against injections, either vaginal or intra-uterine, immediately following labor. His habit is to cover all wounds with iodoform and apply the pad advised by Garrigues, of New York. He has had no bad results from such treatment. In cases of retained secundines, he immediately removes the remaining portions, if need be using force; after which the iodoform and pad are applied.

Dr. Sarah Hackett Stevenson said there are at present two methods of dealing with the secundines—The conservative and the radical. She, in her practice, has used the conservative method, if she has the patient under perfect antiseptic control. The statement of Dr. Earle leaves no room for autogenetic poisoning. It is frequently the case that the patient has within her germs which at the puerperal period infect her without any communication from nurse or doctor. The speaker called attention to the fact that Dr. Ingersoll had neglected to mention the axis-traction forceps of Dr. Bartlett, of Chicago.

Dr. E. P. Cook, of Mendota, thought that the subject was one on which most should agree. He was greatly in favor of an early, complete emptying of the uterus.

Dr. E. Ingals, of Chicago, was not in the habit of using injections. His method is the more conservative one. He relies largely upon nature, considering labor a purely normal process, and the less interference the better.

Dr. J. H. Hollister, of Chicago, spoke in favor of the conservative method of Dr. Ingals. He has used it for thirty-nine years, and his results have been unusually good.

Dr. G. W. Nesbitt, of Sycamore, spoke in favor of the early emptying of the uterus, considering it in every way preferable to any other plan.

Dr. E. Wing, of Chicago, called attention to the difference between asepsis and antisepsis. He also was of the same opinion as Dr. Earle, that there is no such thing as autogenetic infection.

The Society then adjourned to Wednesday at 9 A. M.

WEDNESDAY MORNING SESSION.

The Society was called to order at 9:40 o'clock, Vice-President Wenger in the chair.

The first order of business was the call for voluntary papers, and the following were offered, viz:

Dr. H. Gradle, of Chicago: "On Ocular Disturbances from Nasal Disease."

Dr. F. C. Holtz, of Chicago: "On the Use of the Acetate of Lead in Certain Conditions of the Eye."

Dr. C. W. Earle, of Chicago: "On One Factor in the Etiology, and One Means of Cure in Puerperal Fever."

Dr. S. S. Bishop, of Chicago: "On Operation for Mastoid Disease."

Dr. E. L. Holmes, of Chicago, on "Jequirity."

On motion of Dr. A. Wetmore, of Waterloo, the time for hearing these papers was fixed at after the reception of the reports of committees.

Dr. E. Ingals, of Chicago, read invitations to the Society to visit the Presbyterian Hospital, Rush Medical College, and the Woman's Hospital.

On account of the sudden illness of Vice-President Wenger, Vice-President Ensign took the chair.

The next order being the address of the President, Dr. A. Wetmore, of Waterloo, moved that at any time the order of business shall be suspended to hear the President's address whenever he may be prepared to deliver it. Carried.

Dr. E. Ingals, of Chicago, read the report of the Committee of Arrangements on credentials of delegates and list of members present.

The Secretary read a message of greeting from the Southern Kansas Medical Society, and he was directed to send a return message, in the name of the Society, reciprocating the courtesy.

The resignation of Dr. E. W. Jenks, of Detroit, on account of removal from the State, was accepted.

The receiving of the reports of committees was then proceeded with.

The Chairman of the Committee on Gynæcology, Dr. O. B. Will of Peoria, made an exhaustive report on the progress in that department of the profession during the year. This progress was largely in the way of reconsideration and revision. Special reference was had to the radical change of view concerning the use of certain instruments. The various views held by eminent men concerning pelvic inflammations were fully considered, much attention being given to pelvic abscess. The author believes that thorough evacuation of the pus is the only curative measure in pelvic abscess.

Upon the subject of the removal of the uterine appendages, more perhaps than any other, there prevails a sentiment of distrust and apprehension. No one doubts that the so-called "Tait's operation," or its modification, has been abused. A correct diagnosis is to-day the prominent factor in solving the problem of abdominal section in any given case. One very important symptom in disease of the tubes is frequently a stellicidium of bloody grumous material passed from uterus and vagina, which is regarded by the patient as a prolonged menstrual flow. A further aid to diagnosis may be found in the presence at any time of gonorrhœa. Dr. Howard Kelly relies entirely on bimanual examination. Concerning the technique of the operation nothing new has been developed except the use of hot water within the peritoneal cavity to prevent shock.

In concluding the reporter called attention to the method of tamponing the vagina as recommended by Dr. Englemann, of St. Louis, and Dr. Etheridge, of Chicago.

Dr. Catharine Miller, of Lincoln, of the same committee, reported "Cases of Posterior Misplacement of the Uterus in Virgins, with Comments." These misplacements resemble in most particulars those in parous women, but they differ in some important respects. They are rather frequent. The reporter then gave the histories of three cases which had occurred in her practice during the past winter.

Dr. J. G. Kiernan opened the discussion of the report. He said that on the question brought up by Dr. Miller regarding the influence of local conditions in producing constitutional effects, and also as to the effects of certain habits, such as climbing stairs, rope jumping, and the like, much of importance might be said. It seemed to him, however, that Doctor Miller omitted one condition, and that is this, that a woman coming from a defective family is very likely to have uterine displacements. There has recently been considerable reaction against the excessive use of local vaginal applications in the treatment of conditions like dysmenorrhœa, which appear in a great many cases as the result partly of constitutional disorders.

That dysmenorrhœa is a neurotic disorder there can be no reasonable doubt. In the insane hospitals one finds many female patients suffering from local disorders which are the result of constitutional conditions, and proper treatment of the constitutional condition relieves the pelvic disorder. That the pelvic organs have been much over-treated must be evident to anyone.

He was very much pleased to hear Dr. Miller take the position she does concerning the question of examinations.

Dr. G. W. Jones, of Danville, said regarding the points raised by Dr. Will relative to the danger of pelvic abscess opening into the rectum, he wished simply to record the fact of three cases in his practice that were entirely successful. He wished also to emphasize what Dr. Will had said regarding the use of the dry-wool tampon. He had found it one of the most efficient means for support of the pelvic organs in a large number of cases, particularly in those of relaxation of the soft parts.

Regarding retroversion in virgins, his experience and observation led him to believe that there are a large number of virgins born with retroversion of the uterus, and that it gives them no annoyance. He knows of one family, a mother and five daughters, all of them having retroversion, and all healthy in other respects. In all these cases evidence of the fact that retroversion had existed previous to child-bed was very positive. In virgins a satisfactory examination can be made by careful manipulations through the rectum in eight cases out of ten.

Dr. G. W. Nesbitt, of Sycamore, said he would like to say in regard to the use of the tampon that it seems, from the remarks made by the members, that it is the custom to use the absorbent cotton in making the tampon. The absorbent cotton is very objectionable, because when wet it is very hard. The ordinary fine cotton batting does not pack down as absorbent cotton does. He uses a brand called "Queen's Own." He believes that the original idea of Dr. Sims, in advising the tampon saturated with glycerine, was to use the non-absorbent cotton.

Dr. L. G. Thompson, of Lacon, said he had been very much interested in the papers presented, especially by the practical character of the second one. Regarding Doctor Will's paper, he would say that country doctors do not come across cellulitis and pelvic abscess very frequently. He desired to emphasize one thought in the second paper concerning the importance of considering the liability to uterine trouble in virgins. Many physicians think virgins are not liable to that form of derangement, but his observation led him to believe that it is much more common than many are disposed to think. He finds every now and then a case of uterine displacement accompanied by chronic inflammation in a virgin. In regard to using non-absorbent cotton he would say that when it is removed the next day after it is introduced, it is as good as anything else, but if it is to be left for a longer time, one should use something else. He has been in the habit, where the uterus must be supported, of still applying some form of pessary.

Dr. J. S. Whitmire, of Metamora, said that the idea that a young miss can have congenital retroversion is new to him. It is true that nature may have wonderful caprices, and that certain kinds of exercise in young misses produce inflammation of the uterus, there can be no doubt; and there is no question but that other troubles may be produced in the same way.

Dr. D.T.Nelson, of Chicago, said he was heartily in favor of the suggestions contained in the report. He, however, does not believe in congenital retroversion. He thinks it may be possible, but he regards it as being highly improbable. Tight clothing, high heels and other habits of both mother and child have much to do in producing displacements at a very early age.

Dr. Catharine Miller, in replying to the criticisms on her paper, said she was heartily in accord with many of the suggestions made, and had not embodied some of them in her paper, simply on account of the necessity for brevity.

Dr. O. B. Will said the remarks of Dr. Thompson concerning the country practitioners' not frequently coming in contact with cases of pelvic inflammation, were erroneous. In his mind this class of disorders was first seen by the country practitioner.

Dr. Thompson, in reply, said his remarks related to the country physician and not to the general practitioner in highly-populated centers. He thinks country physicians do not meet frequently with pelvic inflammation.

On motion of Dr. D. W. Graham a recess of ten minutes was taken for the selection of the Nominating Committee.

Dr. S. C. Plummer, of Rock Island, extended an invitation to the Society to hold its annual meeting at Rock Island next year; and the request was referred to Committee on Nominations. The Society then adjourned until 2 P. M.

WEDNESDAY AFTERNOON SESSION.

The Society reconvened at 2:20 o'clock, Vice-President Ensign in the chair.

Dr. D. A. K. Steele gave a brief synopsis of his paper read yesterday afternoon. He also exhibited some of the patients to which he referred yesterday. A case of amputation at the shoulder, in which all the joint tissue was removed, was of special interest.

Dr. P. H. Oyler, of Mt. Pulaski, opened the discussion by inquiring why a compound fracture, or a compound comminuted fracture, cannot be hermetically sealed.

Dr. C. T. Parkes, of Chicago, said he had been much interested in the papers presented by Drs. Steele and Crummer. He had used cocaine in some cases with very happy results, and mentioned one case of ununited fracture in which he cut down upon the bone, and no pain was felt by the patient except when the bone was being divided by the saw. In these cases I do not use the wire in ununited fracture. It has been my experience to cut down upon one or two cases in which wiring had been practised, and in these cases there had remained a fistulous opening, and the cause of the opening seemed to be the wire. He thinks there are times when this method, as well as the use of bone pegs, is advisable, but there are a great many cases of compound comminuted fracture that recover without any such treatment.

Dr. Parkes was unable to understand why Dr. Steele used iodoform to cover the wound surface. Some one has recently said that any form of microbe could be propagated in iodoform. One general principle in surgery is, that a wound surface shall be kept free of any foreign body and iodoform is certainly a foreign body. He reported a case in which suppuration was apparently caused by traumatism, the experiments of Dr. Knapp to the contrary notwithstanding. Dr. Parkes agreed heartily with the portions of the paper concerning examinations of the kidney, and also respecting the necessity of doing the median incision for the exploration of the bladder. He reported the case of a boy in which the operation was the only means of clearing up the diagnosis. Upon introducing the finger into the bladder, a villous tumor as large as the end of a finger was found.

Dr. E. Ingals said the suggestion of Dr. Steele regarding the stretching and falling kidney, reminded him of the the case of a young married lady who had never been pregnant, but consulted him for falling kidney. It was so displaced as to come well down into the abdomen, but could be easily returned to place although it could not be successfully fixed. In the course of time the lady became pregnant and gave birth to a live child at full term; has since had several children, but the kidney became firmly fixed during the first pregnancy and has remained so ever since.

Dr. B. F. Crummer, of Warren, remarked that nothing had been said as to the open treatment of wounds. In his opinion there are certain cases in which the open treatment is best, as, for example, buffer or crushing injuries; better results can be had from suoh treatment han by the cl osing up of the wound.

Dr. D. A. K. Steele, of Chicago, in closing the discussion said he agreed with Dr. Parkes that the wiring and pegging of the bone in many cases is unnecessary. He had also seen some difficulties that Dr. Parkes mentions, as in wire wounds filling with granulations. He said it was his habit to sprinkle a light film of iodoform over the wound surface in amputations, especially if dissolved in ether, as this is an excellent hæmostatic.

There being no further discussion, the report was referred to the Committee on Publication.

On motion of Dr. E. Ingals the report of the Treasurer was read, which showed $1,322.77 in the treasury at the beginning of the present session after satisfying all claims against the Society. The report was adopted and referred to the Committee on Publication.

Dr. Hay moved that the Illinois State Medical Society subscribe $500 toward paying the expenses of the International Medical Congress. The motion was seconded by Dr. Gray, and followed by remarks by Dr. N. S. Davis explaining for what purposes this subscription was needed. After considerable discussion, Dr. Moses Gunn offered an amendment that $750 be subscribed. The amendment was seconded and accepted, and the motion as amended was unanimously carried. This appropriation is the largest thus far made by any state medical society.

The report of the Committee on Drugs and Medicines, Dr. J. G. Tapper, Chairman, was read by Dr. Marie J. Mergler. The paper of Dr. Tapper dealt almost entirely with the prevailing evils existing in the relations between physicians and druggists. He said it is evident to every careful observer that the apparent cordiality existing between the two is quite superficial; that the under-current that has silently flowed on for years has done its work slowly but has at length so deepened and widened the channel, that the position in many localities has become alarming. Looking for the cause of the changed relations, he inquired: "Is it fairly presumable that so large a percentage of physicians are deserting our ranks and becoming pharmacists, that the druggists are becoming alarmed? Is it the terrible annual loss of life as sacrificed by doctors acting in the capacity of dispensers? Both of these questions would be answered in the negative. The skeleton in the closet is a fear that a movement appears to be on foot among many of our reputable physicians of compounding their own medicines and dispensing them directly to patients.

He condemned the practice of refilling physicians' prescriptions, and the almost universal practice of pharmacists exposing for sale their own compounds in the shape of renovators, blood purifiers, dyspepsia cures, etc. According to the belief of the author the prescriptions of a physician are in no way the property of the druggist. Great stress was also laid on the reprehensibility of the substitution of one drug for another, or using different strengths of the same drug. The report closed with suggestions regarding the use of antipyrine in phthisis pulmonalis. The report of Dr. Marie Mergler, "The Progress of Therapeutics in Gynæcology," briefly noticed cocaine and its applications in the treatment of gynæcological cases, and recommends hydrastis canadensis in cases where ergot has usually been given. The author advises iodoform, and more especially an iodoform-gauze dressing, in placing antiseptic douches for operations on the uterus and vagina. According to the experiment of Fellner, hydrastis canadensis is a cardiac poison, producing alteration in blood pressure, and in lower animals produces direct contraction.

Dr. J. H. Hollister, of Chicago, opened the discussion. He considered the points mentioned in the paper of Dr. Tapper of great practical importance. The time has come when there should be some code of ethics or arrangement to govern the relations between physicians and druggists. The matter of re-filling the prescriptions, or substituting drugs, is one for which the medical profession is held responsible; a responsibility which it is not at all anxious to assume. It had occurred to him to carefully prepare a paper, setting forth the relations of the medical profession to manufacturing druggists and dispensing chemists, so fully and clearly that it would be received by both sides as a just exposition of the relations existing.

Dr. A. T. Barnes, of Bloomington, said that to him this had become a very serious question, but that so far as manufacturing chemists were concerned, he could dispose of them. He kept a waste-paper basket in his office to which most of the samples were consigned. Regarding the relations of the druggist and the physician, he would say that in his town it has become the practice for druggists to duplicate a prescription once, twice, or even a hundred times. They have immense books in which they keep a record of prescriptions, and not only re-fill for the patient, but for his family or friends. They have filled a prescription for a certain kind of pills so often that they are called "Barnes' Pills."

Dr. N. S. Davis said there were two aspects to this subject on which his mind had been well settled for a long time. One was, that the physician should adopt the rule, never to prescribe any manufacturers' medicines whatever; and to prescibe no compounds put up by somebody else. The physician should prescribe if he does not use more than one drug, and he should distinctly state what strength of the materials should be used, designating by the United States Pharmacopæia. The second point was, that the dispensing of medicines should be entirely separate from everything else that goes to make up a drug-store. In European countries the dispensers of medicines are generally found in small rooms, the rent of which would not exceed $5 per month. In this country if a person's sickness does not last longer than three days his drug bill will exceed the charge made by the doctor. This system is entirely false ; there is no reason whatever why the sick should pay for marble floors, highly decorated rooms and all the clap-trap that goes to make up the ordinary drug-store. He said he was not complaining against any particular druggist, but against the system which was false from begining to end.

REPORT OF THE COMMITTEE ON OPHTHALMOLOGY AND OTOLOGY.

By the Constitution of this Society it is made the duty of the Standing Committee on Ophthalmology and Otology to "prepare an annual report on all the important improvements in the management of diseases of the Eye and of the Ear, effected during the year."

"Improvement in the Management of Diseases of the Eye," has both a positive and a negative aspect. On the positive side may be classed the advances made in gaining more intimate knowledge of the histology of the eye—its physiology and pathology, as throwing more light on the ætiology of disease as the key to its therapeutics and surgery, since the therapeutics, as well as the surgery, may be regarded as the natural sequence of a demonstrated pathological condition, as the corollary follows from a demonstrated proposition.

Ophthalmic therapeutics, including its materia medica, must be based upon ascertained facts in anatomy, physiology and pathology, and substantial improvement in the management of diseases can only be based upon correct knowledge of the pathological condition with which we have to deal: Correct knowledge of the therapeutic measures or materials used in the treatment of disease, or correct knowledge of the surgical principles involved in the mechanical procedures employed. Since modern, scientific ophthalmology may properly be dated from 1851, when Professor Helmholtz gave to the world the result of the scientific investigation which he and others had been conducting in their efforts to effect a reliable means of examining the interior of the eye during life, an evolution has been going on in ophthalmic science, much of the time with rapid strides. The yearly contributions to the science have been great in all directions, and the result has been an accumulation of varied character, and in a comparatively short time—an accumulation that might almost be regarded as an embarrassment of riches. As a consequence, the negative aspect is forced upon the attention of the investigator in the field who seeks evidence of the true advancement made from year to year.

The process of elimination must be resorted to, and one finds the mistakes that usually attend rapid development and premature announcement of supposed discoveries, and finds that here, as in other places in the wide field of medicine, many cherished theories, many vaunted remedies, and many novel and lauded operative procedures have failed to stand the practical and reliable test of time, and in the necessary culling process have had to be relegated to the list of the disappointing, the useless, or the harmful. The results of such work, for even the past year, could not be embraced in a report such as this is designed to be, nor will the space and the time allotted it permit more than a cursory view of some of the changes wrought in that short time.

Passing over the results of recent investigations of the normal and pathological conditions of the eye, which interest chiefly those engaged especially in ophthalmology, and proceeding to consider the more practical results which may be considered as improvements in the management of diseases of the eye, it may be profitable to consider first the management of the diseases of the appendages of the eye, and later of the eye-ball proper.

In the treatment of inflammation of the conjunctiva, the more heroic measures have been replaced by milder remedies in the acute stages, with great advantages in increased comfort to patients, abbreviation of duration of the affection, and less risk to the integrity of the organ. The introduction of boric acid alone as a topical application to the conjunctiva has done much to simplify treatment of conjunctivitis. The use of cocaine to the conjunctiva continues to meet the expectations aroused by its introduction into ophthalmic practice. Its use in the removal of foreign substances lodged in the conjunctiva or in the cornea, affords great comfort to the patient, and is of real service to the physician in effecting their removal. Another year's trial in the use of infusion of jequirity has served to modify the enthusiastic claims made for it when it was introduced a few years ago, by de Wecker, in the treatment of pannus. Whilst it cannot now be claimed that its use is wholly free from danger, yet the risk is less than that from purulent inoculation of the conjunctiva as practiced years ago, and for which jequirity may be regarded as having in a measure been substituted. Its true value, and its proper place in ophthalmic therapeutics, remain yet to be fully established.

The part which the blennorrhagic micro-coccus or gonococcus plays in producing morbid discharges from mucous surfaces, including the conjunctiva, is one of the problems not yet fully solved.

The recently reported experiments by Professor Knapp, of New York, upon rabbits' eyes have been both interesting and suggestive, and seem to indicate that simple traumatism alone is not sufficient to establish a suppurative process. The germ theory of the origin of disease does not exclude purulent secretion of the conjunctiva. Certainly the locally unirritating parasiticides may be used with advantage in established suppurative processes of the conjunctiva, and asepsis should always be sought for in operations upon the eye or its appendages. New operative procedures are still being proposed, from year to year, for the relief of entropium, but, apparently, with more satisfactory results than attended the earlier efforts. Whilst many of them give temporary relief from the annoyance of that affection, recurrence of the trouble, after a longer or shorter time, seems to be the usual result.

The subject of strabismus and its relief has been receiving renewed attention of late. Beyond the mere mechanical part of the operation of tenotomy, the subject is a diffcult one in many of the cases. Whether the amblyopia which attends many cases be the cause or the consequence, whether it be congenital or acquired, it is a complication and an embarrassment seemingly very difficult of remedy, and it must be confessed that there is yet much to be learned regarding this very common affection.

*Cataract.*—In the operation for soft cataract the needle-operation discision is still the one usually practised. After partial disintegration and softening of the lens, suction is sometimes practised to hasten the removal of the lens substance.

In hard cataract, extraction of the lens is still usually effected by Von Græfe's modified linear extraction, either as suggested by him or as variously modified by others.

Manipulation to hasten the ripening of the cataract, in order to admit of earlier extraction, has not thus far been attended by results which greatly encourage repeated efforts, and its practice is not free from danger.

Incision of the periphery of the capsule of the lens, as suggested for the escape of the lens and to prevent continuance of the nutrition of the capsule, thus to avoid the occurrence of secondary cataract, has not met with general favor.

Scooping-out of the opaque lens in its capsule, with the attendant risk of loss from vitreous body, has commended itself, either on theoretical grounds or by practical results, sufficiently to have led to its general adoption.

The matter of dressing of the eye, called the after-treatment, in operations on the eye-ball, especially in extraction of hard cataract, has attracted considerable attention during the past year, because of great innovations on the long-established custom of bandaging of eyes thus operated upon, and the confinement of patients to dark rooms, with attendant deprivation of accustomed exercise, and under the depressing influence of darkness. The substitution of strips of adhesive plaster over the eye-lids for the heavy bandages so generally used to secure apposition of the edges of the incised cornea and the splint-like support of the eyelids, has given greater comfort and much satisfaction to patients. Thus far no unfavorable reports have been made to indicate that the change in this mode of dressing has resulted otherwise than satisfactory, but it has, probably, not yet been long enough on trial to have determined either its value or the safety of the operating surgeon in case of failure to secure an average result from an operation for cataract, in view of the seeming proneness of many people to seek an excuse for instituting legal proceedings for alleged malpractice in the treatment of eye troubles.

*Glaucoma.*—The varying conditions in acute and chronic glaucoma still continue to be an embarrassment in their treatment, in consequence of want of more accurate knowledge of the pathology of that disease in its early stages; it may safely be alleged that no substitute for iridectomy has yet been discovered that is as reliable as that operation in the treatment of the affection. Eserine and other substitutes cannot give the relief required in the condition called, by Knies, "sclerosing inflammation of the angle of the anterior chamber," with the accompanying and consequent atrophy.

*Sympathetic Ophthalmia.*—This dangerous affection still holds a place, second only in importance to glaucoma, and its ætiology is little if any better understood. Its development seems to be varied in character, and still leaves much doubt as to the avenues of its advance.

The belief formerly held that enucleation of the first affected eye should not be performed after evidence of beginning sympathetic disease in the other eye had manifested itself, is no longer so generally held. A substitute for enucleation of the eye-ball was introduced a few years ago, called opticocitiary neurotomy, and consisted in dissecting the cellular tissue of the orbit, so that by the use of a curved instrument the optic nerve could be divided, and the supposed line of advance of the disease be interrupted. The results which followed a fair trial of the substitute have led to its abandonment.

Evisceration of the globe is a later substitute for enucleation of the eye-ball, in these and other cases in which it is desired to avoid danger of sympathetic disturbance of the unaffected eye, and yet to retain enough of the sclerotic coat of the eye, with its muscular attachments, to afford a moveable support for an artificial eye. It seems to possess merit enough to warrant longer trial.

*Astigmatism.*—Martin (*Annals d'Oculistique*) describes two varieties of astigmatic contractions of the ciliary muscles which tend to neutralize a symnetry of the lens—an astigmatic condition—and makes difficult the adjustment of cylindric glasses; this can only be overcome by strong mydriatics. The same condition complicates latent hypermetropia. In such cases the use of mydriatics is especially indicated, to overcome the spasmodic action of the ciliary muscle and to suspend the accommodative power of the eye, which is a source of embarrassment and uncertainty in adjusting glasses in errors of refraction, and nominally defective accommodative power of the eye.

It is not improbable, however, that, in the absence of such abnormal action of the ciliary muscle, the use of a mydriatic, with its attendant inconvenience and annoyance to patients, is more general than is demanded by the welfare of patients, for repeated testing by trial glasses will enable one to secure the requisite glasses without the use of a mydriatic, and to the great gratification of patients who had previously been subjected to the annoyance of mydriasis for not only days but weeks.

*Plastic Operations.*—Transplantation of conjunctiva from a rabbit's eye to the human subject in cases of loss of a part of that mucous membrane, as by burns and other injuries, has been so successfully done as to have given this plastic operation a probably permanent place in ophthalmic surgery.

The success which attended this operation led to the substitution of the entire eye-ball of an inferior animal, by transplanting it into a human orbit, and with partial success, as to cosmetic effect, in case of loss of the natural eye-ball. If experience should prove that such transplanting of eyes can be substituted for the wearing of artificial eyes. which are more or less of an annoyance, it would prove a great advance in many respects.

Von Hippel has recently reported a successful case of transplanting corneal tissue in the case of a young woman. By removal of an opaque portion from the center of a cornea, by means of a trephine, down to the membrane of Descemet, and then by means of knife and forceps removing still more of the opaque corneal tissue, an opening was made into which he transplanted a piece of cornea from a rabbit's eye, with aseptic precautions. Rapid union took place; for a few days the transplanted portion was cloudy, but at the end of a week it had become transparent again, and when report of the case was made eight months later the transparency continued with indications that it would be permanent.

Vision through the transplanted portion was reported as being $\frac{20}{200}$ or 1-10.

Dr. A. E. Prince, of Jacksonville, in his report called attention to an instrument for regulating the pressure used in inflation of the middle ear. It is impossible to arrive at an estimate of the damage done by the unregulated force employed in the indiscriminate use of the politzer bag in the treatment of middle ear diseases. To facilitate the method of inflation and diminish its dangers, attention is called to a graduated mercurial column upon which the patient is required to blow. The instrument is essentially a re-curved tube filled with mercury and supplied with rubber tube against which the patient is required to blow while the ears are inflated. While the mercury is deposed the air is driven by the bag through the nostril in the usual way, and, a force indicated by the pressure of the mercury, plus the momentum of the blast, is expended in inflating the middle ears, and any surplus force, in place of working an over-distension of the drums simply overcomes the upward pressure of the palate, and escapes into the pharynx. This instrument Dr. Prince calls a politzometer.

The report of the Committee on Necrology was read by the Chairman, Dr. E. Ingals, of Chicago. It embraced notices of the late President, Dr W. T. Kirk; of Dr. James S. Jewell; Dr. R. C. Hamill and Dr. McArthur.

The Society adjourned to 9 A. M. Thursday.

### THURSDAY MORNING'S SESSION.

The Society was called to order at 9:45 o'clock, Vice-President Ensign in the chair.

The special order being the address of the President, Dr. E. P. Cook, in behalf of the President, gave a short abstract of the address, and it was referred to the Committee on Publication. The address gave a retrospect of the progress of medicine in the State of Illinois during the past fifty years.

The report of the Committee on Publication, Dr. James F. Todd, Chairman, was read by Dr. D. W. Graham.

Three hundred and fifty copies of the Transactions for 1886 were published at a cost of $348.96. About 60 copies remain at present in the hands of the Secretary, subject to the disposal of the Society. The report was accepted.

The report of the special Committee on Diseases of Children was read by the Chairman, Dr. George Wheeler Jones, of Danville. In preparing his report the Doctor sent the following questions to two or more physicians in each county:

First—"Has there been much sickness amongst the children in your county or district during the past year?"

Second—"What have been the prevailing diseases, and what has been the general type of such affections?"

Third—"Have you had in your district any outbreaks of any of the contagious or infectious diseases of children?"

Fourth—"Have you used, in the treatment of children's disorders, any new line of management seeming to give more favorable results than other plans previously adopted?"

Fifth—"Do you believe that the State Legislature ought to appropriate annually a sum of money sufficient in amount to defray all expenses connected with investigations covering the subjects embraced in 'Infant Mortality: Its Causes and Reduction,' and 'The Contagious Febrile Diseases: Their Prevention and Cure'?"

About twenty-five per cent. of the reports represented an increase of sickness amongst children. The counties in the southwestern portion of the State, and in the low bottom lands of the Mississippi valley, and those representing the lower altitudes of the interior were most involved in increase of children's disorders. Excepting the contagious and infectious diseases, the general line of disease consisted in bowel complaints last summer and respiratory disorders during the winter, all more or less influenced by malarial toxæmia. An unusual prevalence of rheumatism characterized the sickness of the past winter.

With but one exception every reporter mentioned the presence of one or more of the contagious or infectious diseases. The epidemic influence has thus been general throughout the state, but as a rule in a mild form. Scarlet fever, german measles, diphtheria and pertussis have been especially prevalent. The majority of the cases of german measles have lacked the post auricular glandular enlargement which has been considered almost pathognomonic. It seems that rheumatism has never been so prevalent in both old and young as now. For this disease Dr. Jones places great reliance on the phospho-iron preparations and on salicine.

In the reporter's opinion the "avitreous thermometer," is the best for use with children. The report closed with a short review of the therapy of children's diseases.

In response to the last question on the list, concerning legislative appropriations, the answer of ninety per cent of the correspondents was "yes."

Dr. F. J. Shipp, of Petersburg, contributed to the report a paper on

THE CARE OF CHILDREN.

In the first place great stress was laid on cleanliness, and, equally important in the new-born, no water bath shall be used during the first 24 hours of extra-uterine life. The child is given a thorough oil bath, lightly covered with some suitable material and at the expiration of twenty-four hours a warm bath of very short duration is given in a warm room. The report dealt also with causes of disease and finally the diseases to which children are liable.

Dr. E. Ingals, of Chicago, in opening the discussion of Dr. Jones' paper, said there was one point in relation to the treatment of pertussis to which no allusion had been made and that was the use of chloral and morphia. It is a much more simple treatment than any other. The speaker now uses practically nothing else. It is especially effective in the later stages. He uses one thirty-second of a grain of morphia and one grain of chloral, frequently repeated if necessary in, a child of three years. Concerning the treatment of the diarrhœas of children he endorsed the antiseptic treatment. He was much in favor of rest and laxatives. He has discarded astringents in such cases. It may be useful in some cases to use opiates to relieve irritation but not as astringents. His plan is to empty the bowel and leave it alone.

Dr. Robert Tilley said there was one point mentioned in Dr. Jones' paper to which reference had before been made, and that was the matter dignified by the name of Skeer's symptoms. He knew but little about the symptoms, but had made an effort to get all the knowledge obtainable. When the report was brought up before the Pathological Society of Chicago, a committee was appointed to investigate this particular symptom, and at that time one case was referred to which was thought to be typical. It was at St Luke's Hospital, but upon inquiry at the hospital it was learned the case was not a typical one and had left the hospital. This, however, was the only case that the committee had an opportunity to investigate. Dr. Tilley thought too much importance had been attached to the little "Skeer's symptom." Of course in a well-established peculiarity like the Argyll-Robertson symptom it is well enough to have a name applied; but it would be in bad taste to hoist this supposed peculiarity on the medical profession without a great deal more evidence than we have at present.

Dr. H. N. Moyer, of Chicago, in replying to Dr. Tilley, said that inasmuch as he wrote the report referred to he might be responsible for the term "Skeer's symptom." At the time when the reference was made the committee did not have any cases of tubercular meningitis under their care, but they took every means to get at a case. There were doubtful cases of tubercular meningitis. In these cases the symptoms was not present. The case that was seen in St. Luke's Hospital was declared by Dr. Skeer to be typical in appearance. In Dr. Skeer's paper a distinction is made between a symptom somewhat resembling the "Skeer symptom" seen in scrofulous children and that found in tubercular meningitis, which undergoes changes in a few hours. Dr. Moyer took great pains to look up the literature. Nothing could be found. He communicated with Dr. Block, and he had never seen the condition or read of it, but concluded from the description and drawings sent him that it must be due to an inflammatory exudate which subsequently became colored by the coloring matters of the blood, thus accounting for the varying color changes. He further thought that these changes were not due to any specific infection but rather to intracranial pressure. The pathology is very doubtful, and until it could be definitely settled it was decided to term the condition Skeer's symptoms.

Dr. C. W. Earle, of Chicago, said that in this city there had been a great number of cases of measles, and an epidemic of german measles; also a few cases of varicella, the severity of which he had never witnessed before There has been a great tendency shown to occurrence of a second attack In a large number of cases of german measles post auricular glandular enlargement has been absent. This has made him doubtful as to where to place the cases spoken of.

Dr. A. H. Foster, of Chicago, said his experience had been that of a rather typical epidemic of measles. Following the cases of measles, in three to six weeks there were many cases of german measles without the post auricular enlargement. In several of these cases a longer stage of convalescence was present. This he attributed to the debility arising from the first attack.

Dr. S. W. Nesbitt, of Sycamore, had seen many cases of german measles, sometimes one or two members in the family having the first auricular glandular enlargement, while in others of the same family it was absent. This, he thought, proved that german measles could cccur without the enlargement spoken of by Dr. Earle.

Dr. J. H. Hollister, of Chicago, thought the physicians of his city had seen more cases of repeated measles and more german measles than ever before during the history of the city.

Dr. E. L. Herriott, of Jacksonville, said that cases of german measles seen in his region last year were very light, but thls year the cases have been very severe and the epidemic quite pronounced.

Dr. A. E. Goodwin, of Rockford, had seen many cases during they ear of german measles; very little treatment was necessary. He wished to know whether there was any law requiring children to have a physician's certificate before returning to school after an attack of measles?

Dr. George Wheeler Jones, of Danville, in closing the discussion, remarked that he heartily indorsed the rules laid down by Dr. Shipp concerning the treatment of the new-born, especially the oil bath. He had tried chloral and morphia, but on account of its depressing effect had ceased to use it. He had seen many cases of german measles without the post auricular enlargement, and had been somewhat at a loss where to classify them.

The report was referred to the Committee on Publication.

The special report on physiology, by Dr. A. Wetmore, of Waterloo, was passed over on account of the absence of the author.

THE REPORT OF THE NOMINATING COMMITTEE

was read and adopted, as follows:

President—William O. Ensign, of Rutland.

First Vice-President—Chas. Warrington Earle, of Chicago.

Second Vice-President—Philip H. Oyler, of Mount Pulaski.

Permanent Secretary—David W. Graham, of Chicago.

Assistant Secretary—G. L. Eyster, of Rock Island.

Treasurer—Walter Hay, of Chicago.

Judicial Council—G. A. Wetmore, of Waterloo; S. C. Plummer, of Rock Island; and B. F. Crummer, of Warren.

Committee of Arrangements—C. Truesdale, Thos. Galt, C. Bernhardt, G. G. Craig, S. C. Plummer, C. C. Carter, Geo. E. Barth, P. Gregg, all of Rock Island.

Next Place of Meeting—Rock Island.

STANDING COMMITTEES.

Committee on Practice of Medicine—Frank Billings, of Chicago; G. W. Nesbitt, of Sycamore; A. H. Taggert, of Chicago.

Committee on Surgery—S. W. Caldwell, of Freeport; B. F. Crummer, of Warren; S. K. Crawford, of Chicago.

Committee on Obstetrics—George Wheeler Jones, of Danville; C. C. Hunt, of Dixon; Catharine Slater, of Aurora.

Committee on Gynæcology—A. R. Jackson, of Chicago; T. M. Cullimore, of Jacksonville; Mary E. Bates, of Chicago.

Committee on Drugs and Medicines—D. S. Booth, of Sparta; A. J. C. Saunier, of Libertyville; J. A. Robison, of Chicago.

Ophthalmology and Otology—E. L. Holmes and S. S. Bishop, of Chicago; C. A. Palmer, of Princeton.

Necrology—E. Ingals, of Chicago; J. A. Rauch, of Springfield; O. B. Will, of Peoria.

Committee on Publication—D. W. Graham and Walter Hay, of Chicago; G. L. Eyster, of Rock Island.

SPECIAL COMMITTEES.

Diseases of Children—A. E. Goodwin, of Rockford; Mary H. Thompson, of Chicago; W. West, of Belleville.

Antiseptic Obstetrics—Charles Warrington Earle, of Chicago.

Intubation of Larynx—F. E. Waxham, of Chicago.

The Present Status of Bacteriology in Its Relation to Disease—R. J. Curtis, of Joliet, and F. A. Johnson, of Chicago.

Pelvic Surgery—W. J. Chenowith, of Decatur, and Sarah Hackett Stevenson, of Chicago.

Venereal Diseases—H. N. Moyer, J. N. Danforth, and J. J. M. Angear, all of Chicago.

Hypnotics—D. R. Brower, of Chicago.

Compound Fractures—D. A. K. Steele, of Chicago.

Hernia—D. W. Graham, of Chicago.

Physiology—A. Wetmore, of Waterloo.

Dermatology—H. J. Reynolds, of Chicago.

Neurology—H. Wardner, of Anna, and J. L. Gray, of Chicago.

The Exigencies of Excitements During the First Labor—J. S. Whitmire, of Metamora.

Some Topic on Gynæcology—Marie J. Mergler, of Chicago.

Legislation for the Insane—Walter Hay, of Chicago; Edgar P. Cook, of Mendota; Francis B. Haller, of Vandalia.

Medical and Sanitary Legislation—B. M. Griffith, of Springfield; W. A. Haskell, of Alton; John L. White, of Bloomington; Albert B. Strong, of Chicago.

Influence of Appreciable Meteorological and Topographical Conditions on the Prevalence of Acute Diseases—Nathan S. Davis, of Chicago; John H. Hollister, of Chicago; James F. Todd, of Chicago; Edgar P. Cook, of Mendota; George W. Jones, of Danville.

Biographical—John H. Hollister, of Chicago; Ephraim Ingals, of Chicago; Charles C. Hunt, of Dixon; Francis B. Haller, of Vandalia; A. M. Powell, of Collinsville; Edgar P. Cook, of Mendota; John S. Williams, of Otterville; George W. Jones, of Danville; Thomas F. Worrell, of Bloomington; Robert Boal, of Peoria; Hugh R. Guthrie, of Sparta.

To Promote the Organization of Local Medical Societies throughout the State—Edgar P. Cook, of Mendota.

Dr. E. P. Cook read a supplementary report as follows:

This Committee, composed of one member from each county represented, deem it not beyond their province to call the attention of the society to other matters connected with its best interests. We consider it unwise to close our eyes to the fact that the past meetings have failed to bring out the attendance that it should have and to develop the interest that this Society, representing over 3,000 regular practitioners in this State, should possess. Why this is so, we are not prepared to state, but we feel that the future existence of this Society rests on the action taken on this subject in the near future. What measures should be taken to improve the attendance at and interest in our meetings are now matters of very serious consideration, and this must be had at once. In view of these facts we would present the following:

"*Resolved*, That a live working committee of three members be appointed to consider this subject, and such committee be authorized to have the results of their deliberations printed in circular form and mailed to each member. This committee shall have the power to suggest such amendments to the Constitution as they shall think best adapted to attain the ends desired. In addition such committee shall prepare and mail to every regular physician in this State such information concerning this Society as shall be necessary to enlist their cooperation in our State Society work."

T. M. McIlvaine.
E. P. Cook.
A. E. Goodwin.

On motion of Dr. J. H. Hollister the rules were suspended and Dr. E P. Cook, of Mendota, presented the report of the Committee to Promote the

ORGANIZATION OF LOCAL MEDICAL SOCIETIÉS THROUGHOUT THE STATE.

"In our judgment the subject is of such importance as to require the appointment of a committee to whom shall be assigned the duty, under instructions and by authority of the State Society, to open a correspondence with one or more of the leading members of the profession in each county in the State where no medical societies now exist, and urge upon them the importance of medical organization—and representation—of the profession of all parts of our State in the State Medical Society. We further recommend that this duty be assigned to the same committee suggested by the Nominating Committee.

Dr. J. H. Hollister, of Chicago, said that the history of medicine in Illinois had in the past been creditable. We are now at a point where a large number of young men should be enlisted in the work. Just such a committee is needed.

Dr. D. W. Graham, of Chicago, said that the constitution and by-laws are in an exceedingly crude form and a committee to consider suitable amendments is an absolute necessity.

On motion of Dr. H. N. Moyer the recommendations were adopted and the President authorized to appoint a committee of three.

The President announced as such committee E. P. Cook, of Mendota; Thomas McIlvaine, of Peoria; and A. E. Goodwin, of Rockford.

On motion of Dr. E. C. Cook, the nomination of delegates to the American Medical Association for 1887 was proceeded with, and the following persons elected:

E. B. Loomis, Edward Pynchon, Moses Gunn, E. Ingals, J.L.Gray, J. E. Owens, H. J. Reynolds, A. R. Reynolds, S. K. Crawford, J. M. Hall, Walter Hay, D. W. Graham, H. H. Frothingham, Norman Bridge, J. J. M. Angear, and Denslow Lewis, of Chicago; C. C. Hunt, Dixon; William O. Ensign, Rutland; Thomas McIlvaine, Peoria; E. A. Kilbourne, Elgin; W. S. Caldwell, Freeport; F. L. Nutt, Marengo; K. E. Rich, Winona; G. W. Nesbit, Sycamore; S. E. Plummer, Jr., Rock Island; E. A. Ingersoll, Canton; D. S. Jenks, Plano; B. F. Crummer, Warren; David Prince, Jacksonville; A. Nash, Joliet; E. P. Catlin, Rockford; R. Boal, Peoria; A. K. Van Horn, Jerseyville; W. M. Sweetland, Highland Park; W. M. Barrett, Onarga; E. P. Herriott; Jacksonville; Washington West, Belleville; H. T. Hardy, Kaneville; R. W. Gillett, Carlinville; N. B. Cole. Bloomington; A. J. C. Saunier, Libertyville, and J. A. Freeman, Millington.

On motion of Dr. E. Ingals, of Chicago, the secretary was empowered to fill vacancies.

Dr. C. W. Earle, of Chicago, moved that the President appoint a committee of three to name delegates to the American Medical Association for 1888. Carried; and the President named C. W. Earle and D. W. Graham, of Chicago, and G. W. Jones, of Danville.

On motion of Dr. Norman Bridge, of Chicago, Dr. B. F. Uran, of Kankakee, was made a member by invitation.

Dr. D. W. Graham, of Chicago, asked for an appropriation of $25 to secure and bind a complete set of transactions of the Society. Carried.

The Society then adjourned to 2 o'clock p. m.

THURSDAY AFTERNOON'S SESSION.

The Society was called to order at 2 o'clock, Vice-President Ensign in the chair.

Dr. H. J. Reynolds read the report of the Special Committee on Dermatology, calling attention to the treatment of lupus vulgaris by electrolysis, after the method of Gartner and Lustgarten. The flat negative pad is placed over the lupous nodule, a sufficient current to produce escharotic effect is used, and the wound treated after the usual manner.

In urticaria a 2 to 10 per cent. solution of menthol is advised. It is equally effective in pruritis, where camphor solutions are not well borne. In acue of the female, the hot vaginal douche is said to have relieved several cases. The action of cocaine upon the skin if introduced by the cataphoric action of the galvanic current is mentioned as being entirely successful in producing local anæsthesia.

On motion of Dr. E. Ingals, the Biographical Committee was continued, with power to add to its numbers.

Dr. S. S. Bishop, of Chicago, read a paper on "Operations for Mastoid Disease," the object of which was to increase confidence in operations on the mastoid process. In all cases of accumulation of pus in the mastoid region, free incisions are made and the cavity rendered thoroughly aseptic. In those cases where necrosis exists, the diseased bone is removed by chise- or curette. The report concluded with the details of treatment in seven cases occurring in the author's practice during the year.

On motion, the report was referred to the Committee on Publication.

Dr. E. F. Ingals, Chairman of the Committee on Diseases of the Throat and Nose, read a paper on epistaxis, with a recital of cases from his own practice. Epistaxis may be simply the result of a local plethora. In old people the most frequent causes are degenerative changes in the blood vessels. The treatment of the disorder is of two kinds, that designed to check the hæmorrhage and that to prevent its recurrence. For the former, one of the most effective measures is firm compression of the nostrils for 10 or 15 minutes. The application of cold is equally efficacious. In more aggravated cases syringing out the nose with cold water and the subsef quent insufflation of a powder containing 4 per cent. of cocaine and a suitable proportion of iodoform is highly recommended. The author's method o plugging the nares is as follows: A piece of surgeon's gauze, an inch in width and four feet in length, is thoroughly saturated in a solution of tannia prepared by rubbing with sufficient water to make a thin sympy mixture. Opening the nostril with a speculum, one end of the slip is folded on itself and over the end of a probe, by which it is carried quickly to the posterior part of the nasal cavity, and then fold after fold is passed slowly in until the cavity is completely filled. This plan has been successful in extreme cases of epistaxis.

Dr. R. Tilley, of Chicago, opened the discussion. He doubted the statement made by the author that hæmorrhage occurring from the nose is usually an effort of nature to relieve plethora. It is rather an evidence of a weak point in the nose, and the physician should try to discover it and apply a remedy. Polypi are not infrequent in the young, and when present it should be as truly removed as when in an adult.

Dr. L. H. Montgomery, of Chicago, gives preference to a solution of the dialyzed tincture of iron used with absorbent cotton. It has in his hands been very effective.

Dr. H. A. Johnson said that in young persons about the period of puberty there is an excess of blood over that needed for the nutrition of the individual. At such times there is a tendency to hæmorrhage from surfaces poorly supported by surrounding tissues. The speaker does not place much reliance on nitrate of silver in such cases. The Poquelin cautery is much more effective.

Dr. N. S. Davis, of Chicago, gave a verbal report of the Committee on the "Influence of Appreciable Meteorological and Topographical Conditions on the Prevalence of Acute Diseases." The Committee was continued.

Dr. Walter Hay, of Chicago, gave a verbal report on "Legislation for the Insane." The report was discussed by Drs. Kiernan, Cook, Hollister, Bridge and Davis; and the following resolution, offered by Dr. Bridge, was adopted:

"*Resolved*, That this Society heartily indorses the provisions of the bill now before our legislature providing for the commitment of the insane in certain cases without trial by jury; and asks the legislature to enact these provisions at least."

On motion of Dr G. W. Jones, the rules were suspended, and Dr. E. P. Cook presented the following resolution:

"*Resolved*, That the thanks of this Society are hereby extended to the Chairman of the Committee of Arrangements and all who have in any way contributed to making this thirty-seventh annual meeting a success."

Dr. G. W. Jones, of Danville, gave notice that at the next annual meeting a resolution would be introduced changing the wording of Article VII of the Constitution by substituting the words "two dollars" for the words "three dollars."

Dr. C. W. Earle, of Chicago, gave notice that an amendment to Article V would be introduced at the next annual meeting, adding the words "a Committee on Diseases of Children, and a Committee on Neurology" to paragraph 1 of said Article V.

On motion of Dr. S. K. Crawford, Dr. H. Z. Gill, of El Dorado, Kan., was made an honorary member of the Society.

The Society adjourned to meet at Rock Island the third Tuesday in May, 1888.

